# The Birmingham Mandible and Mid-face (BruMM) rules: interim data analysis

**DOI:** 10.1308/rcsann.2024.0107

**Published:** 2025-07-15

**Authors:** ROC Elledge, S Vatharkar, D McNulty, D Parekh

**Affiliations:** University Hospitals Birmingham NHS Foundation Trust, UK

**Keywords:** Zygoma, Facial fracture, Mandible, Radiograph

## Abstract

**Introduction:**

Clinical predictor rules are useful heuristics that can inspire confidence in clinicians on the front line to make decisions that are safe and reproducible. Rules such as the Ottawa Ankle Rules can also reduce the number of unnecessary radiographs taken, reducing radiation exposure and cost, as well as improving quality of care.

**Methods:**

A previous Delphi study delineated 11 variables associated with an increased likelihood of finding a mandibular fracture and 14 variables associated with an increased likelihood of finding a zygomatic fracture on plain film radiographs. In the current study, clinicians suspecting a mandibular and/or zygomatic fracture were invited to complete a proforma identifying any of these variables in advance of requesting plain film radiograph(s). An interim analysis was conducted with predictors being cross-tabulated against relevant outcomes using: sensitivity, specificity, Jaccard index, odds ratio (OR) and Fisher's exact probability.

**Results:**

During the period January to October 2022 inclusive, 69 records were inputted into REDCap, of which 20/69 demonstrated a fracture. Fisher’s exact test produced several significant results including malocclusion (*p*<0.0001, OR 31.99), presence of a new open bite (*p*<0.001, OR undefined) and subconjunctival haemorrhage (*p*<0.05, OR undefined).

**Conclusions:**

Data collection is continuing and initial interim analysis would suggest a sample size of *n*=252 will be required, assuming a negative scan rate of 55%, to achieve a specificity of 0.90 within ±0.05. We aim to present finalised data in 2025.

## Introduction

The premise of the Birmingham Mandible and Midface (BruMM) Rules Study is to develop a maximally sensitive clinical predictor rule for the likelihood of facial fractures on plain film radiographs. It is apparent that maxillofacial emergencies, particularly facial trauma as a result of interpersonal violence, may represent a disproportionate number of attendances at emergency departments.^1^ Despite this, given the breadth of knowledge required in Emergency Medicine, physicians charged with diagnosis and management at the front line may lack the confidence and ability to deal with these.^2,3^ For this reason, heuristics and algorithms can ensure safety, maximise efficiency and inspire confidence in clinicians.

Precedents exist in creating maximally sensitive predictor rules for reducing the number of unnecessary radiographs, most notably the Ottawa Ankle Rules used by emergency departments around the world.^4^ Although some work has been done previously on the determination of the likelihood of facial fractures on plain film radiography, the created rules often fail to achieve high specificity, and variable determination for inclusion has been questionable.^5,6^

The first author has previously published a Delphi study to determine optimum variables for inclusion in the development of a facial fracture predictor algorithm for mandibular and midface fractures (the so-called Birmingham Mandible and Midface, or BruMM, Rules). This was three successive waves of questionnaires submitted to Members/Fellows of the British Association of Oral and Maxillofacial Surgeons (BAOMS).^7^ The purpose of the current work was to apply these variables in determining a correlation with the likelihood of facial fractures on plain film radiography.

## Methods

**Figure 1:**
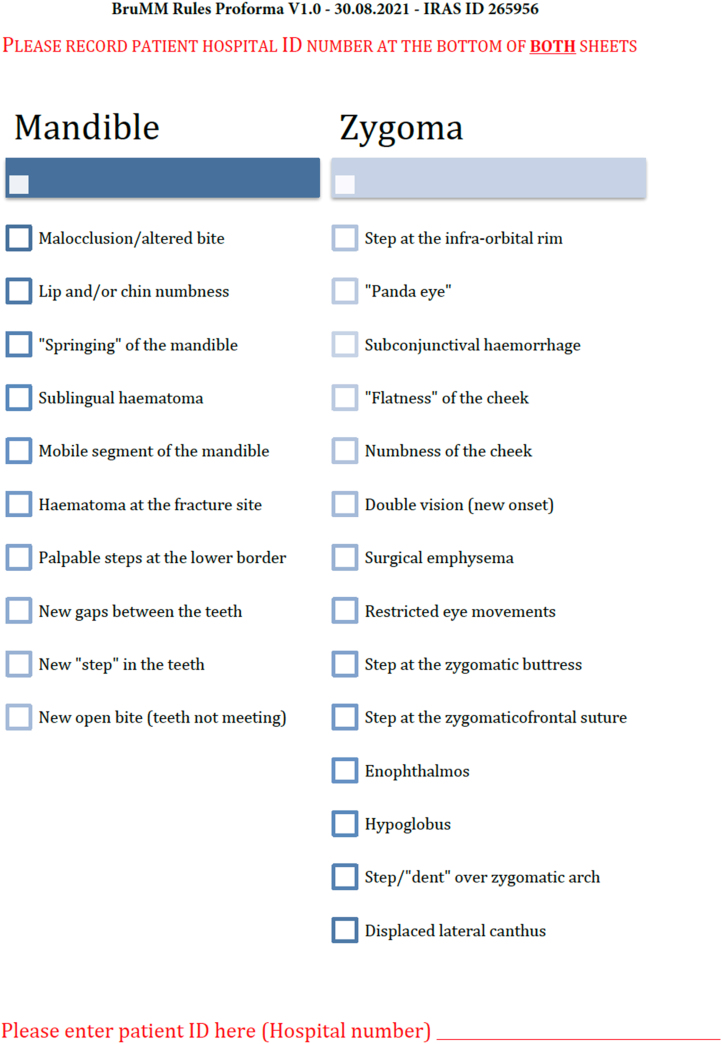
BruMM Rules proforma v1.0 used for data collection and based on previous Delphi study. BruMM = Birmingham Mandible and Midface

**Figure 2:**
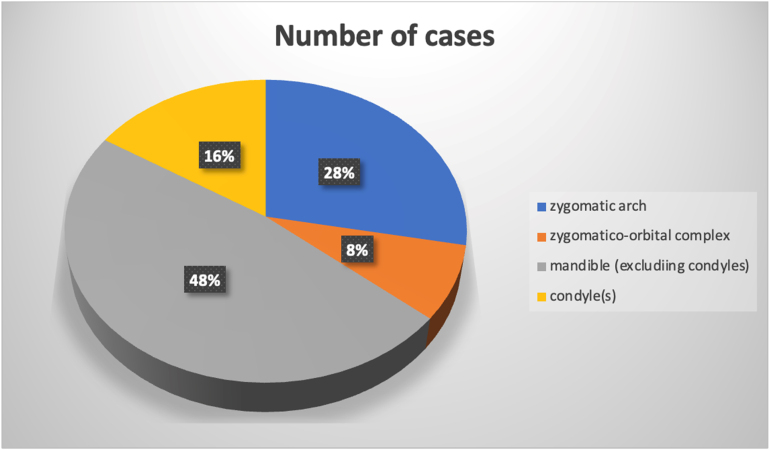
Fractures by subtype within interim data analysis

The resulting Delphi study informed the development of the proforma included below (Figures 1 and 2). Ethics approval was obtained for the study (UHB R&D reference RRK6797/IRAS Project ID 265856/NHRA Research Ethics Committee (REC) Approval 19/NW/0587) with a favourable opinion granted by the NHS Health Research Authority (HRA) and Health and Care Research Wales (HCRW) on 15 October 2019. A substantial amendment was submitted and approved on 28 July 2020. Category C amendments were approved on 4 November 2021 and data collection for this interim analysis continued up to 8 October 2022.

Members of a dedicated BruMM Rules Study Group were recruited from the emergency departments at Queen Elizabeth Medical Centre, Good Hope Hospital and Birmingham Heartlands Hospital, all part of University Hospitals Birmingham NHS Foundation Trust. The premise of the study was explained using online tutorials as either live events or prerecorded access-on-demand resources.

BruMM Rules Study Group members were charged with championing the project in their respective emergency departments and collecting any completed BruMM Rules proformas, uploading the data to REDCap and transferring paper proformas to designated secure storage areas. All members were registered with EDGE and REDCap and asked to provide a current curriculum vitae and Good Clinical Practice certificate for central storage.

**Figure 3:**
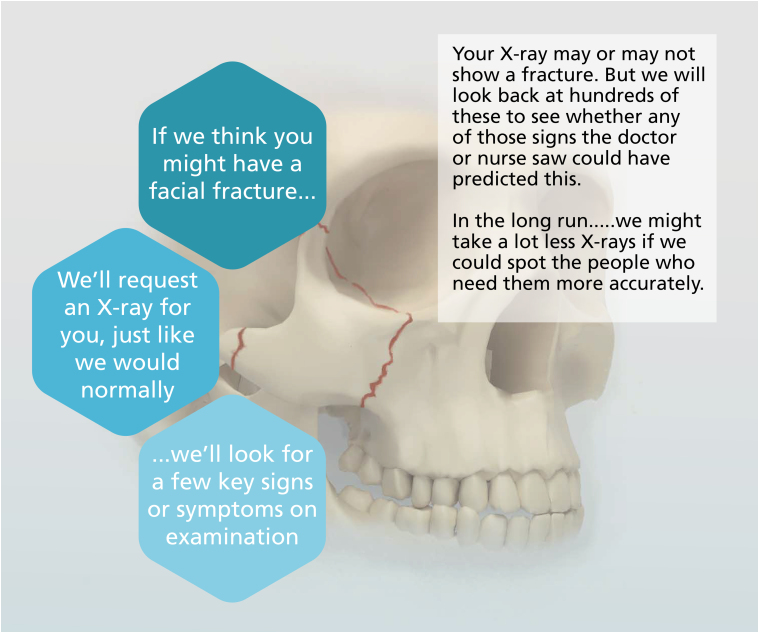
Poster displayed in all clinical areas to inform potential study participants, enabling opt out decision

In line with the terms of the HRA/HCRW REC approval, posters regarding the study were displayed in all waiting areas of the emergency departments to inform patients of the study and to enable patient to ‘opt out’ (Figure 3).

Any frontline clinician seeing a patient with presumed facial fractures deemed to require plain film radiograph(s) as the investigation of choice was asked to complete a BruMM Rules proforma in advance of requesting their radiograph(s) (Figure 1). The need to complete the proforma was highlighted as in no way questioning the judgment of the clinician, which could not be superseded, and it was made clear at the outset that even were none of the suggested variables identified, the clinician could still request the radiograph if their clinical judgment dictated that they would have otherwise done so. The following exclusion criteria applied for patients being recruited to the study:

• Edentulous patients
• Patients requiring computed tomography and/or magnetic resonance imaging as their initial imaging modality (e.g. isolated orbital fractures, panfacial and/or polytrauma patients)
• Patients with previous facial fractures

Radiographs were then reviewed by BruMM Rules Study Group members and any fractures identified were stratified as either:

• Mandible fractures (excluding condyles)
• Mandibular condyle fractures
• Isolated zygomatic arch fractures
• Zygomatico-orbital complex fractures

The objective is to construct indicators that have a high false negative rate (specificity) to reduce the number of negative radiographs. It is assumed indicators posited by the Delphi study are sensitive, specifically the combined set of indicators are sufficiently exhaustive having near 100% sensitivity, that sample size may be determined by measuring specificity to a known tolerance/precision (Table 1).

**Table 1:** Sensitivity and specificity results from interim data analysis

	Facial fractures					
Indicator	Sens	Spec				
Any mandible	65.0%	79.2%				
Any zygomatic	35.0%	45.8%				
	Mandibular condyle fractures		Other mandibular fractures		Any mandible	
Indicator	Sens	Spec	Sens	Spec	Sens	Spec
Any mandible	100.0%	70.3%	91.7%	78.6%	92.3%	80.0%
Any zygomatic	0.0%	48.4%	0.0%	41.1%	0.0%	40.0%
	Isolated zygomatic arch fractures		Zygomatico-orbital complex fractures		Any zygomatic	
Indicator	Sens	Spec	Sens	Spec	Sens	Spec
Any mandible	14.3%	63.9%	50.0%	66.7%	14.3%	63.9%
Any zygomatic	100.0%	57.4%	100.0%	53.0%	100.0%	57.4%

Sens = sensitivity; Spec = specificity

Specificity is number of true negative predications (TN) divided by the number of negative fracture scans (TN+FP), where FP is the false positive count. Assuming TN and FP counts have Poisson distributions, specificity has a binomial distribution with probability=specificity, and sample size=negative fracture scans.

The initial sample size is determined by solving $\mathrm{Tolerance}=1.96\sqrt{\mathrm{Spec}(1-\mathrm{Spec})/n}$ for *n*. The final sample size is determined by dividing *n* by. The final sample size is determined by dividing *n* by the proportion of negative scans. Table 2 illustrates a range of sample sizes using different parameters.

**Table 2:** PPV and Jaccard from interim data analysis

	Facial fractures					
Indicator	Jaccard	PPV				
Any mandible	43.3%	56.5%				
Any zygomatic	14.9%	20.6%				
	Mandibular condyle fractures		Other mandibular fractures		Any mandible	
Indicator	Jaccard	PPV	Jaccard	PPV	Jaccard	PPV
Any mandible	17.4%	17.4%	45.8%	47.8%	50.0%	52.2%
Any zygomatic	0.0%	0.0%	0.0%	0.0%	0.0%	0.0%
	Isolated zygomatic arch fractures		Zygomatico-orbital complex fractures		Any zygomatic	
Indicator	Jaccard	PPV	Jaccard	PPV	Jaccard	PPV
Any mandible	3.4%	4.3%	4.2%	4.3%	3.4%	4.3%
Any zygomatic	20.6%	20.6%	5.9%	5.9%	20.6%	20.6%

PPV = positive predictive value

True Positive (TP), False Negative (FN), FP and TN counts, may be viewed as Poisson distributed counts conditional on the total sample size. Given two Poisson random variable *X* and *Y* with means $\lambda_{X}$ and $\lambda_{Y}$ the ratio Z=X/(X+Y) has a Binomial distribution with probability $p=\lambda_{X}/(\lambda_{X}+\lambda_{Y})$ and count n=X+Y.

The mean μ and variance $\sigma^{2}$ and a Binomial distributed random variable *R* with probability *p* and sample size *n* are μ=np and $\sigma^{2}=p(1-p)/n$ respectively. Providing the smaller of np and n(1−p) is at least five, the asymptotic normally distributed confidence intervals for the proportion r/n are $r/n\;\pm z_{1-\alpha /2}\sqrt{r(n-r)/n^{3}}$, where $z_{1-\alpha /2}$ is the standard normal deviate with probability 1−α/2.

An interim analysis was conducted with predictors being cross-tabulated against relevant outcomes using: sensitivity, specificity, Jaccard index and odds ratio (OR). Any calculation using a count of five or less was suppressed.

## Results

In the study period to 1 October 2022, a total of *n*=69 records were inputted into REDCap, of which 20/69 demonstrated a fracture, translating to 71.0% of cases having radiographs unnecessarily. Fracture subtypes were as demonstrated in Figure 2 (five patients had a combination of fracture types).

Interim results demonstrate proof of principle with Delphi mandibular indicators having sensitivity 92.3% and specificity 80.0% to mandibular fractures and zygomatic indicators having sensitivity 100% and specificity 57.4% to zygomatic fractures. Results should be viewed with caution due to the small number of cases (specifically 20 fractures from 69 cases) and the imbalance of mandibular to zygomatic fractures. A target specificity of 90% having 5% tolerance where approximately 55% of samples are negative requires approximately 252 cases.

## Discussion

Clinical predictor rules continue to be developed and published in the wider literature, with recent examples including predictors for sacral fractures, cervical spine fractures and scaphoid fractures.^8–10^ The step beyond a heuristic for the clinician is machine learning, and big datasets enable deep learning by artificial neural networks, enabling them to develop optimum models through representation learning.^11^ Convolutional neural networks can deal with grid-structured topological data, including images, surpassing the accuracy of clinicians and making diagnostics more predictable, accurate and efficient.^12^ At the current time, artificial intelligence (AI) in Radiology is focused on fracture detection and outcome prediction in the main, rather than the prediction of the specific likelihood of fractures based on clinical parameters.^13,14^ There are emerging precedents for fracture risk prediction in specific contexts (e.g. identifying patients at risk of osteoporotic fractures),^15^ and it may be conjectured that AI may have a role in conjunction with heuristics such as the BruMM Rules to facilitate decision-making for the clinician.

The selling point of the Charalambous model^5^ was maximal sensitivity. On reflection, a 100% sensitivity may not be an appropriate measure of success in designing a predictor rule. The frontline doctor who is tired and will request scans ‘just to be safe’ may lead to a high rate of FNs. Outcomes may also be skewed when clinicians exercise hubris, and refuse to consider the possibility of missing a fracture, raising their threshold for radiograph requests with false confidence. As yet, at the interim analysis of the BruMM Rules Study, the sample size is too small but indications exist that a composite measure will have high sensitivity and specificity.

The Ottawa Ankle Rules were devised in 1992 using 750 patients and 32 variables and employed the recursive partitioning methodology.^16^ This may not be the ideal multivariate analysis method as there is the potential for ‘overfitting’ of the data (whereby the model fits too closely to the specific data, thereby failing to predict future observations) and methodologies such as random forest analysis.^17^ This is based upon bootstrap aggregating^18^ (‘bagging’) whereby replacement training sets are selected randomly from the data to fit decision trees to different samples. Prediction for unseen samples *x*′ is then averaged from predictions of all trees:

$$\hat{\;f}=\frac{1}{B}\overset{B}{\underset{b=1}{\sum }}f_{b}(x^{\prime })$$

where *B* is the number of times bagging is performed, *f_b_* is the regression tree and *x*′ is unseen samples. Decision trees mandate that all variables hold true in the cascade. Potentially in the current project, a composite measure of ‘any of’ the variables holding true may show superior accuracy, sensitivity and specificity.

Moving forwards in terms of statistical analysis, predicated on a larger sample size, the indicators will be modelled separately and in combination where indicators are combined using logical OR operations. Predictors that are poorly populated may be combined using logical OR operations subject to clinical approval. The numbers in this interim data analysis were small, in part due to issues in achieving ‘buy in’ from clinicians on the ‘shop floor’ in the emergency department with completing proformas, as well as publicising the study in house to gain traction in recruitment. Incorporating Emergency Medicine physicians into the BruMM Rules Study Group, regular attendance at teaching days and engagement of Emergency Nurse Practitioners (more likely to see these minor trauma patients) are among the strategies being employed in maximising participant recruitment.

BruMM Rules will be constructed in two steps. The initial step will construct a predictive score. The best predictive score model will then be simplified to a BruMM Rule by constructing an indicator equal to the logical OR of any predictor in the predictive score.

Predictive scoring models will be calibrated on a two-thirds sample with internal cross-validation. Model building strategies prior to final construction of the BruMM Rules are left to the discretion of the analyst and may include, among other techniques, logistic regression and tree methods. The selected BruMM Rules will be validated on the remaining third of the sample. This will ultimately enable the development of a clear simplified final algorithm for adoption in clinical practice, which is the endpoint goal of the BruMM Rules study.
